# Is TWEAK a Biomarker for Autoimmune/Chronic Inflammatory Diseases?

**DOI:** 10.3389/fimmu.2013.00489

**Published:** 2013-12-27

**Authors:** Daniel Bertin, Delphine Stephan, Michel Khrestchatisky, Sophie Desplat-Jégo

**Affiliations:** ^1^Aix-Marseille Université, NICN, CNRS, UMR7259, Marseille, France; ^2^Service d’Immunologie, Pôle de Biologie, Hôpital de la Conception, Assistance Publique – Hôpitaux de Marseille, Marseille, France

**Keywords:** TWEAK, biomarker, auto-immunity, disease monitoring, serum levels, urinary levels

## Abstract

The TWEAK/Fn14 pathway is now well-known for its involvement in the modulation of inflammation in various human autoimmune/chronic inflammatory diseases (AICID) including lupus, rheumatoid arthritis, and multiple sclerosis. A panel of data is now available concerning TWEAK expression in tissues or biological fluids of patients suffering from AICID, suggesting that it could be a promising biological marker in these diseases. Evidences from several teams support the hypothesis that blocking TWEAK/Fn14 pathway is an attractive new therapeutic lead in such diseases and clinical trials with anti-TWEAK-blocking antibodies are in progress. In this mini-review we discuss the potential use of TWEAK quantification in AICD management in routine practice and highlight the challenge of standardizing data collection to better estimate the clinical utility of such a biological parameter.

## Introduction

Autoimmune and chronic inflammatory diseases (AICID) including rheumatoid arthritis (RA), multiple sclerosis (MS), systemic lupus erythematosus (SLE), or systemic sclerosis (SSc) constitute an important medical, social, and economic problem. The prevalence of AICID is estimated to be more than 3% in the adult population (1) and despite scientific progress in the past decade, identification of new reliable markers for diagnosis, prognosis, and prevention of hospitalization is still necessary.

TWEAK is a pleiotropic and multifunctional cytokine that regulates inflammatory pathways by inducing multiple cellular responses depending on the cell type and its micro-environment. During tissue repair and remodeling, the biological activity of TWEAK is complex and even dual: after acute injury, TWEAK promotes tissue regeneration especially by stimulating progenitor cells, but in chronic diseases where TWEAK is persistently activated, it alters tissue repair in part by inhibiting differentiation of the same progenitor cells (2). A growing body of data points to the involvement of the TWEAK/Fn14 pathway in inflammation in various human AICID including SLE, RA, and MS. It is now admitted that TWEAK plays a role in the physiopathology of such diseases and the first clinical trials are in progress, based on anti-TWEAK-blocking therapies during RA or SLE (3, 4). Nevertheless it remains to be proven that the assessment of TWEAK levels in tissues or biological fluids is of interest for the management of patients suffering from AICID. In this article we propose to review the available data on TWEAK quantification in human AICID and to discuss the potential place and the modalities of TWEAK evaluation in the diagnosis and/or the follow-up of AICID.

## TWEAK Evaluation in AICID: Why, When, and What for?

Biomarkers in medicine have gained immense scientific and clinical interest in recent years. Biomarkers are potentially useful in the context of primary, secondary, and tertiary prevention. An ideal biomarker should be safe to assess, easy to measure and associated with acceptable costs. Additionally, the biomarker should have “good performance characteristics” (i.e., sensitivity, specificity, positive- and negative-predictive values) and there should be scientific evidence to suggest that biomarker modification influences disease outcome.

In AICID, various serum circulating auto-antibodies are widely used as reliable markers for diagnostic or sometimes prognostic evaluation. Nevertheless the identification of new markers to evaluate inflammatory activity of the disease, to predict disease flare or to monitor the clinical response to biotherapy remains necessary.

The idea that TWEAK could be such a biomarker in AICID is suggested by its role in the modulation of inflammation in AICID both in animal models and in human pathologies. Moreover, in the past decade, various research groups have shown modulation of TWEAK expression in tissue and biological fluids of patients suffering from AICID.

### For establishing a diagnosis of AICID

Very few data are available concerning the diagnostic potential of soluble TWEAK in AICID. Among them, urinary TWEAK (uTWEAK) has been proposed by Schwartz and colleagues as a biomarker for lupus nephritis (LN) as they showed that it is elevated in subjects with LN at diagnosis compared with those with SLE but no renal disease (5). Moreover uTWEAK levels were better at distinguishing LN and non LN-SLE than anti-DNA antibodies and complement levels. Schwartz et al. also reported that serum TWEAK (sTWEAK) levels and the urine/sTWEAK ratio were not better markers for LN than the less invasive uTWEAK. It is intriguing to note in this work that sTWEAK levels were significantly lower in SLE patients than in healthy individuals. Later, El-Shehaby et al. have also shown that uTWEAK levels positively correlate with renal involvement during SLE with a positive predictive value of the marker of 93% for kidney inflammation (6).

### For following-up disease activity

Autoimmune and chronic inflammatory diseases are chronic diseases that usually fluctuate between periods of remission (little/no symptoms) and unpredictable flare-ups (worsening symptoms). In this context, the ability to predict the severity of disease and the outcome of flares should allow tertiary prevention of disease complications. In LN, renal biopsy remains the gold standard for assessment of disease activity but because of its invasive modalities, this approach cannot be used repeatedly in clinical practice to follow-up disease activity. In this context, uTWEAK has been shown to correlate with the degree of clinical disease activity during LN (5) and fluctuations of uTWEAK levels were found to reflect LN flares. uTWEAK could thus represent an interesting biological tool if the diagnosis of a flare is in doubt.

In RA, auto-immunity and chronic inflammation leads to the destruction of cartilage and bone in the joints. The relationship between TWEAK and arthritis has been supported by anti-inflammatory and anti-angiogenic effects of TWEAK inhibition in a mouse model of collagen-induced arthritis (7, 8). Park et al. showed for the first time that sTWEAK levels were higher in RA patients than in healthy controls (9). Moreover, sTWEAK levels in RA patients positively correlated with the DAS 28 disease activity scores (9).

In 2009, Yanaba et al. described for the first time elevated sTWEAK levels in patients suffering from another AICID, SSc, a generalized connective tissue disorder characterized by sclerotic changes in the skin and internal organs. The longitudinal study of sTWEAK levels performed in this study showed that these high soluble TWEAK levels were associated with a lower prevalence of pulmonary fibrosis and better pulmonary function in SSc patients. This perturbed expression of TWEAK suggested some involvement of the TWEAK pathway in the appearance of pulmonary fibrosis, a well-known complication of the disease. Nevertheless the mechanisms and the meaning of the elevation of sTWEAK levels in this context remain to be determined (10).

### For monitoring therapies targeting TNF superfamily members

Currently, few autoimmune diseases can be cured with treatment. In the last decade, TNF blocking agents have been developed for the treatment of human diseases and have been very successful in ameliorating disease signs and symptoms especially in patients suffering from AICID like RA.

According to the work of Park et al. high sTWEAK levels have been shown to correlate with TNF alpha levels in RA patients and to reflect short-term response to etanercept, a biotherapy targeting TNF alpha (9). In fact, responders to etanercept showed a significant decrease in sTWEAK levels at the 12th week of treatment, whereas TWEAK levels in non-responders were not different from their baseline levels. TWEAK is a member of TNF family. Like TNF inhibition, TWEAK inhibition is an attractive therapeutic option in AICID. The results of the first study exploring the safety and tolerability (phase I) of a TWEAK-blocking therapy using the monoclonal antibody BIIB023 in patients with RA have been recently published (3). They demonstrated that a single-dose administration of BIIB023 was well tolerated in patients with RA and was associated with a suppression of sTWEAK levels for up to 28 days in the case of BIIB023 high dose. The efficacy of BIIB023 (phase II) is now explored in the ongoing clinical trial ATLAS (Anti-TWEAK in Lupus Nephritis patients) for patients with lupus nephritis, a pathology that requires a more innovative treatment than RA. In this context, quantification of soluble TWEAK in anti-TWEAK treated patients is an interesting way for managing BIIB023 administration.

## TWEAK Evaluation in AICID: How to Proceed?

### Which form of TWEAK to quantify: membrane and/or soluble form?

TWEAK expression has been studied in various AICID animal models and in humans (11–14). The main cellular sources of TWEAK are cells from the monocyte/macrophage family including immune infiltrating cells in tissues during inflammation. TWEAK can expressed as a type II transmembrane protein or efficiently cleaved to generate sTWEAK. Elevated levels of sTWEAK can be found locally at the site of diseased tissue during AICID. The presence of membrane-bound TWEAK has also been described in human cells (15–17) but not in other studies of freshly isolated peripheral blood mononuclear cells (PBMCs) from normal subjects or patients with RA or SLE (Linda Burkly, personal communication). Nevertheless the relevance of one form versus the other is not yet elucidated. Modulation of the soluble form levels rather than the membrane form is more frequently studied and published.

### Which sample to collect and to use for TWEAK quantification?

Soluble TWEAK is currently quantified in patient serum due to the facility of routine blood collection by venous puncture. But depending on the target tissue involved in AICID soluble TWEAK could be quantified in various other body fluids such as synovial fluid (18), cerebrospinal fluid (17), or urine (19) and may better reflect inflammatory activity in respectively the joint, the brain, and the kidney. TWEAK expression during AICID has also been studied in PBMCs by western blot and PCR. In SLE, elevation of TWEAK in PBMC is correlated with disease activity and lupus nephritis (20). In SSc, the production of soluble TWEAK by cultured PBMC is significantly diminished in patients with more severe microvascular damage as indicated by capillaroscopy (21). In the context of neuroinflammation, Serafini et al. reported strong TWEAK expression on post-mortem MS brain tissue while TWEAK was generally undetectable by immunohistochemistry in the normal human brain white matter or cerebral cortex samples (11). Even if these results are very interesting for further evaluation of the role of TWEAK in MS physiopathology, these approaches are not appropriate for routine diagnosis of MS. Desplat-Jégo et al. have shown by using PBMC analysis after culture that TWEAK is expressed at the cell surface of circulating monocytes during MS and not during non-MS neurological inflammatory diseases (17). This study also showed that TWEAK was detectable in the serum and CSF of MS patients but found no significant difference between the MS group and other clinical groups.

In RA, data on TWEAK/Fn14 expression in the synovium of patients are available (12, 18) and support a high level of TWEAK in RA synovial tissue concordant with the numerous activated macrophages found in synovium during the disease and with the beneficial effect of TWEAK blockade in a mouse model of RA.

### To quantify TWEAK alone or associated with other(s) biological parameter(s)?

Panel-based approaches measuring a combination of biological parameters are more and more used for routine biological diagnosis of human diseases. Very few studies have addressed the diagnostic performance of associating TWEAK with one or several other markers.

Moreno et al. have reported that soluble CD163/soluble TWEAK ratio in peripheral blood is a more sensitive biomarker of subclinical atherosclerosis than soluble CD163 or soluble TWEAK alone (22). CD163 is a member of the scavenger receptor cysteine-rich family now recognized as an immunomodulator of the atherosclerotic plaque. It has been previously proposed that TWEAK could specifically bind and neutralize TWEAK and that CD163-expressing monocytes/macrophages were able to bind and internalize exogenous TWEAK (23). However this interaction has recently failed to be confirmed (24). Nonetheless, Kowal-Bielecka et al. have recently shown that a high serum soluble CD163/soluble TWEAK ratio is associated with lower risk of digital ulcers but with a more severe skin disease in patients with SSc (25).

In the case of spot urine exploration of soluble TWEAK, it is recommended to normalize TWEAK levels to urinary creatinine measured in the same spot urine since creatinine excretion rate into the urine is relatively constant and allows to compensate for the variability in the volume of urine and the concentrations of soluble TWEAK from void to void (5).

## TWEAK Evaluation in AICID: How to Define Pathological Values?

In the first place, normal value ranges have to be determined prior to the definition of pathological values for sTWEAK levels in humans. A threshold for pathological TWEAK levels has to be established. It is not an easy task. Different ranges of normal values issued from various laboratory teams are available (Table 1) and there is obviously a lack of homogeneity. The differences observed in soluble TWEAK levels in healthy control subjects depend presumably on various parameters: age, sex, ethnic origin of the recruited control group, pre-analytical conditions especially sample storage conditions before analysis, the technical characteristics of the ELISA kits, nature of TWEAK epitopes recognized by anti-TWEAK antibodies, capacity of soluble TWEAK to trimerize etc. Table 1 summarizes the data obtained by 10 teams and shows the difficulty in obtaining to date consensual normal values for sTWEAK levels.

**Table 1:** **“Normal” mean values and ranges for serum soluble TWEAK in humans determined by ELISA**.

Publication reference	ELISA kit manufacturer	Number of control healthy subjects tested	Serum soluble TWEAK levels (pg/ml) Mean ± SD (ranges)
Park et al. (9)	Bender Medsystems	40	42.7 ± 14.5
Yanaba et al. (10)	Bender Medsystems	31	41.5 ± 33.5
Yilmaz et al. (26)	Bender Medsystems	55	445 (326–634)
Chorianopoulos et al. (27)	Bender Medsystems	30	377 (310–432)
Xia et al. (28)	Bender Medsystems	45	34.8 (15–54)
Kowal-Bielecka et al. (25)	Bender Medsystems	48	294 ± 147 (132–1015)
Turkmen et al. (29)	Bender Medsystems	25	457 (320–538)
Desplat-Jégo, personal results	Bender Medsystems	58	467 (200–866)
Schwartz et al. (5)	Home-made ELISA	19	23.5 (17.3–27.1)
Llaurado et al. (30)	R&D Systems	68	1636 (1146–3754) for men and 1401 (788–2422) for women

*SD, standard deviation*.

In urine, LN patients have been compared with control subjects but data obtained in this latter group was detailed by only one team in 23 healthy volunteers with a reference value for uTWEAK of 5.67 pg/mg of creatinine (interquartile range: 3.10) (5, 6, 19).

In the case of other biological fluids (CSF, synovial fluid) data are lacking to establish normal ranges especially because collections of such fluids are invasive and not allowed in control healthy subjects by ethic committees.

## In Conclusion

On the one hand, quantification of uTWEAK in AICID seems to be an interesting tool in the follow-up of disease activity especially in lupus nephritis. On the other hand it is premature to propose sTWEAK as a diagnostic tool of AICID due to the lack of specificity for one disease in particular. Moreover studies analyzing diagnostic performance of serum sTWEAK are still needed. sTWEAK assay is nevertheless useful in the follow-up of anti-TWEAK biotherapies and perhaps could be useful in the follow-up of anti-TNFα therapies but this latter point needs to further be evaluated. At this point, the most important criticism concerning TWEAK evaluation in AICID is the difficulty in obtaining consensual ranges for normal values of sTWEAK. Reagents for the assessment of TWEAK levels are now commercially available for research purposes only. However, standardization of the proposed kits is absolutely required all the more because free soluble TWEAK is a “sticky” protein difficult to manipulate based on experience from our team and others (31). Moreover comparison between studies using different reagents must be conducted carefully. It is our opinion that the standardization of data collection is critical to further evaluate the clinical utility of TWEAK as a promising biomarker.

## Conflict of Interest Statement

The authors declare that the research was conducted in the absence of any commercial or financial relationships that could be construed as a potential conflict of interest.
